# Human Case of Lobomycosis

**DOI:** 10.3201/eid1004.030416

**Published:** 2004-04

**Authors:** Sameer Elsayed, Susan M. Kuhn, Duane Barber, Deirdre L. Church, Stewart Adams, Richard Kasper

**Affiliations:** * Calgary Laboratory Services, Calgary, Alberta, Canada; † University of Calgary, Calgary, Alberta, Canada; ‡ Calgary Health Region, Calgary, Alberta, Canada

**Keywords:** Lobomycosis, Lobo’s disease, Loboa loboi, Lacazia loboi, infection, human, keloidal blastomycosis

## Abstract

We describe a 42-year-old woman with histologically confirmed lobomycosis, a cutaneous fungal infection rarely reported outside of Latin America. Our case represents the first published report of imported human lobomycosis in Canada and the fifth in an industrialized country.

In February 2001, a healthy, 42-year-old, female geologist from Canada came to her dermatologist with a slowly growing, 1.5-cm diameter, dusky-red, nontender, plaque-like lesion surrounded by keloidal scar tissue on the posterior aspect of her right upper arm (Figure 1). It was located at the site of a scar from a previous excision attempt of a similar lesion 2 years earlier. The original lesion was first noticed while the patient was visiting Southeast Asia in 1996, although she did not seek medical attention until returning to Canada 1 year later. At that time, coccidioidomycosis was diagnosed based on her history of previous travel to a lobomycosis-endemic region and on the presence of oval, yeast-like organisms in histologic sections. However, *Coccidioides immitis* was never cultured from the lesion, and serologic studies for this fungus were negative. After the excision in 1997, nothing was noted further until October 1999, when a small lesion, similar in color to the original one, reappeared under the scar and gradually increased in size. The patient had no other skin lesions and was otherwise asymptomatic.

**Figure 1:**
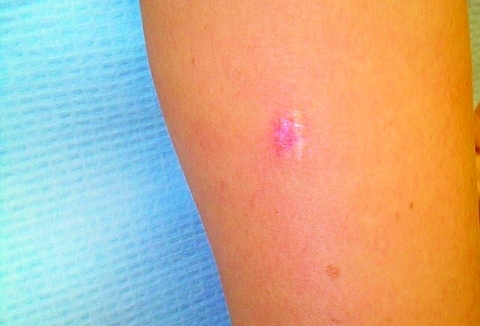
Photomicrograph of keloidal skin lesion on patient’s right upper arm.

The patient had spent time doing geologic work in various tropical regions over a 7-year period. In 1992–1993, she traveled to the Four Corners region of the United States as well as to California, northern Mexico, and Costa Rica. She lived and worked in the jungles of Guyana and Venezuela for 2 years (1993 and 1994), spending most of her time around the Cuyuni, Essequibo, and Rupununi River areas, although she also spent some time in the Bolivar state of Venezuela. Thereafter, she traveled to Kazakhstan, Indonesia (Irian Jaya), and the Philippines (1995 to 1996). During her time in South America, she lived mainly in rural camps and had extensive exposure to freshwater, soil, and underground caves. Health problems encountered during her travels included dengue fever, amebic dysentery, and intestinal helminthiasis. Of note, she had never traveled to the African continent.

Her medical history was otherwise unremarkable. Family history was positive for hypothyroidism. She was a nonsmoker and a social alcohol drinker. She was on hormone replacement therapy but no other medications and had no known allergies. Review of systems was unremarkable. Other than the lesion on her right arm, results of physical examination were normal.

Biopsied tissue specimens of the lesion were obtained and submitted for pathologic and microbiologic examination. The hematoxylin- and eosin-stained tissue sections showed a diffuse, superficial, and deep granulomatous dermatitis with multinucleated giant cells. Intracellular and extracellular unstained fungal cells with thick refractile walls were seen, giving a “sieve-like” pattern to the granulomatous inflammation. The fungal cells stained strongly with periodic acid-Schiff and Grocott methenamine silver stains (Figure 2); cells were spherical or lemon-shaped, approximately 10 μm in diameter, and uniform in size. They were arranged as single cells or in short budding chains joined by narrow, tubelike bridges. Calcofluor white stain (Figure 3) of fresh tissue indicated fluorescent, spherical fungal organisms similarly arranged in chains. The organisms were not cultivatable. Fungal morphology was consistent with *Loboa loboi*. The lesion was completely excised with no subsequent recurrence.

**Figure 2:**
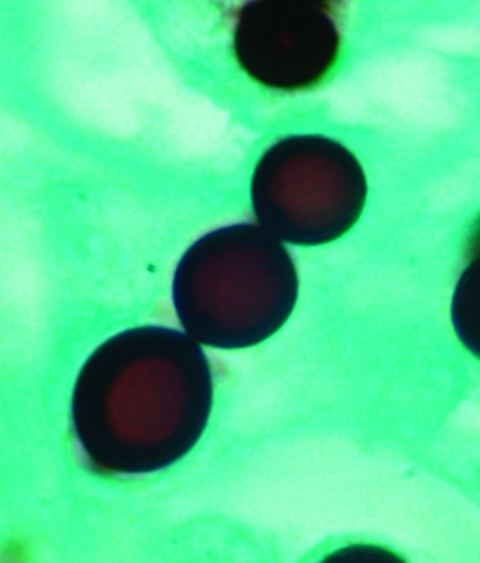
Grocott methenamine silver–stained section of skin biopsy specimen revealing a short chain of lemon-shaped fungal cells connected by thin, tubelike bridges. Morphology is consistent with the appearance of *Loboa loboi*. Magnification x1,000.

**Figure 3:**
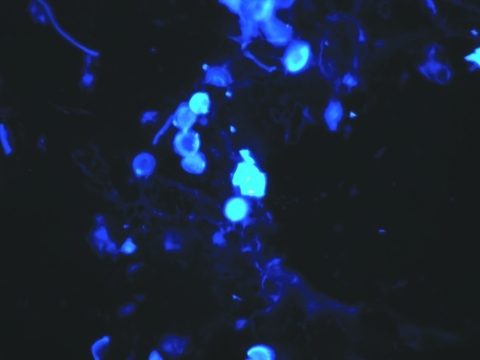
Calcofluor white stain of skin biopsy specimen. A short chain of lemon-shaped fungal cells is seen in the left-center of the field. Cells are connected to each other by thin, tubelike structures. Magnification x400.

## Discussion

Lobomycosis is a chronic granulomatous infection of the skin and subcutaneous tissues caused by the fungus *L.* *loboi* (*1*,*2*). It is characterized by the appearance of slowly developing (months, years, or decades), keloid-like, ulcerated, or verrucous nodular or plaque-like cutaneous lesions (*1*,*2*), usually at a site of local trauma such as from a cut, insect bite, animal bite, or ray sting (*1*–*3*). Lesions may be single or multiple and tend to occur on exposed, cooler areas of the body, particularly the extremities and ears (*1*,*2*,*4*). Other sites such as the forehead, face, chest, scapula, lumbosacral spine, buttocks, and scrotum have also been reportedly involved (*2*–*7*). Mucous membranes or internal organs are not involved (*2*,*3*). Lesions may be nonpigmented, hypopigmented, or hyperpigmented and are usually painless or only slightly pruritic (*1*,*3*,*4*). Little if any local host inflammatory response and no systemic symptoms exist (*3*,*4*). In some cases, lesions may spread contiguously or through lymphatic channels, causing cosmetic disfigurement (*2*,*4*,*5*).

On histologic examination, the lesions are composed of dermal granulomas with multinucleated giant cells filled with spherical or lemon-shaped fungal cells 6–12 μm in diameter with doubly refractile walls that are commonly arranged in chains of budding cells connected by thin, tube-like bridges (*1*,*2*,*6*,*8*). *Loboa loboi* has never been cultivated in vitro (*1*,*2*), although it has been successfully transmitted to armadillos, tortoises, and the footpads of hamsters and mice under experimental conditions (*2*,*6*).

Natural disease has been described only in humans and in marine and freshwater dolphins (*1*,*2*). Lobomycosis was first described by the dermatologist Jorge Lobo in 1931 (*2*–*4*,*9*). His patient was a 52-year-old man who worked as a rubber collector in the Amazonas state of Brazil, who had numerous slowly developing nodular keloidal lesions in the lumbosacral spine area, from which microorganisms resembling *Paracoccidioides brasiliensis* were observed on microscopy (*2*). Lobo suspected that the patient had a modified form of paracoccidioidomycosis, which he called keloidal blastomycosis (*2*). A second human case was reported in 1938, after which the disease was termed Lobo’s disease (*2*). More than 500 human cases have been reported to date (*3*), although the disease appears to be confined to remote tropical areas of South and Central America, especially in communities along rivers (*1*–*3*). The natural habitat of the fungus is not known but is believed to be aquatic or associated with soil and vegetation (*2*,*3*). Rubber workers, farmers, miners, fishermen, and hunters are particularly at risk due to extensive outdoor exposure (*2*–*4*). The condition has been described in Bolivia, Brazil, Colombia, Costa Rica, Ecuador, French Guiana, Guyana, Mexico, Panama, Peru, Suriname, and Venezuela (*2*–*4*).

The first report of nonhuman infection occurred in 1971 in an Atlantic bottle-nosed dolphin from Florida (*10*). In 1973, a dolphin with Lobo’s disease was described in Europe along the Atlantic coast of France and Spain (*11*). The dolphin’s caretaker, a resident of Holland, later acquired the disease, the first human case of lobomycosis reported outside of Latin America (*11*). Other cases of lobomycosis in dolphins have been confirmed, including one in a dolphin off the Texas coast (*2*,*12*).

Cases of imported human lobomycosis have been described in the United States and elsewhere (*6*,*7*,*13*,*14*). A case of lobomycosis involving the chest was recently described in an Atlanta, Georgia, man who had previously traveled to Venezuela and was exposed to extremely high water pressure while walking under Angel Falls (*7*), although the first published description of a human case in the United States appears to be in an immigrant from Suriname (*6*). Recently, cases of imported human lobomycosis were reported in France (*13*) and Germany (*14*). Other imported cases in industrialized countries are believed to occur but may be misdiagnosed due to physician unfamiliarity with the disease. As far as we are aware, our report is the first of human lobomycosis in Canada. The disease was presumably acquired in Guyana or Venezuela, because her visits to Mexico and Costa Rica were unlikely to put her at risk for infection.

Based on our patient’s history, the physical findings, and the histologic appearance of the skin lesion, the diagnosis of lobomycosis was unequivocally made. Although other fungi may resemble *L. loboi* microscopically, including *P. brasiliensis*, *Blastomyces dermatitidis*, and *Histoplasma capsulatum* var *dubiosii* (the cause of African histoplasmosis), none of them form the characteristic chains of fungal cells of uniform size, 6- to 12-μm in diameter, connected by thin tubelike isthmuses, the hallmark of lobomycosis (*8*). Furthermore, unlike *L. loboi*, the other fungi can be grown in vitro on routine mycologic media (*2*,*8*). In contrast to *L. loboi*, the mother cell of *P. brasiliensis* forms multiple buds and remains larger than the daughter cells, giving the characteristic “ship’s wheel” appearance (*1*,*15*,*16*). In addition, paracoccidioidomycosis is a disease of the oronasal mucous membranes and lungs (*1*,*16*). In contrast to *B. dermatididis*, the fungal cells of *L. loboi* do not form broad-based buds (*8*). Because the patient had never traveled to Africa, she was not at risk for African histoplasmosis. Other conditions that may clinically resemble lobomycosis include keloids, leprosy, leishmaniasis, chromoblastomycosis, and malignancy (*2*,*4*).

Lobomycosis does not usually affect the general health of a person. However, unless lesions are removed at an early stage, the disease persists for life (*2*). Rarely, squamous cell carcinoma may develop from lobomycotic lesions (*2*,*17*).

Successful treatment of lobomycosis usually consists of total surgical excision of the lesion, preferably with wide margins (*1*,*2*,*9*), although adjunctive medical therapy with clofazamine or other agents has sometimes been used in patients with extensive disease, with limited success (*2*). However, a German patient with lobomycosis had complete clinical and histopathologic resolution of disease after a 1-year treatment course of oral clofazamine and itraconazole (*14*). One dolphin with Lobo’s disease was successfully treated with miconzaole (*2*). Our patient has remained disease-free for more than 2 years after surgical excision of her lobomycotic lesion.

The nomenclature of the fungus has been subject to ongoing debate, although a new genus, *Lacazia*, with *Lacazia loboi* as the type species, was recently proposed by Toborda et al. (*16*) and appears to be taxonomically valid. The definitive taxonomic status of this fungus awaits in vitro culture and subsequent molecular studies, although phylogenetic analysis of 18S small-subunit rDNA molecules indicates that *L. loboi* is a distinct and novel species phylogenetically close to but fundamentally different from *P. brasiliensis* (*15*).

In summary, lobomycosis is a slowly progressive, chronic, fungal infection of the dermis that is rarely seen in industrialized countries. This disease should be suspected in patients with single or multiple keloidal skin lesions, particularly if they have traveled to remote areas of Latin America.
